# Sex-specific effect of cortisol on cerebral glucose metabolism across Alzheimer's disease spectrum: a neuroimaging study

**DOI:** 10.3389/fneur.2026.1680116

**Published:** 2026-03-12

**Authors:** Abdullah Alqarni, Essam Alkhybari, Mohammed S. Alshuhri, Manal Aljuhani, Ibrahim Hadadi, Manal H. Alosaimi, Hussein Alshaari, Mansour M. Alqahtani, Amr A. Abd-Elghany, The Alzheimer's Disease Metabolomics Consortium

**Affiliations:** 1 Department of Radiology and Medical Imaging, College of Applied Medical Sciences, Prince Sattam bin Abdulaziz University, Al-Kharj, Saudi Arabia; 2 Department of Radiological Sciences, College of Applied Medical Sciences, King Khalid University, Abha, Saudi Arabia; 3 Department of Radiological Sciences, College of Applied Medical Sciences, King Saud University, Riyadh, Saudi Arabia; 4 Radiological Sciences Department, College of Applied Medical Sciences, Najran University, Najran, Saudi Arabia; 5 Biophysics Department, Faculty of Science, Cairo University, Giza, Egypt

**Keywords:** Alzheimer's disease, brain hypometabolism, cerebral glucose metabolism, cortisol, FDG-PET, healthy aging, MCI, sex differences

## Abstract

Higher levels of cortisol can disrupt the normal patterns of cerebral glucose metabolism in the human brain. This study aims to investigate the effects of elevated cortisol levels on cerebral glucose metabolism in men and women across Alzheimer's disease spectrum. The data was derived from the publicly available Alzheimer's Disease Neuroimaging Initiative (ADNI) database. Eight hundred and twenty-two participants across varying diagnostic cognition status were included: 469 men (mean age of 74.14 ± 7.19 years) and 353 women (mean age of 72.31 ± 7.34 years). Main effect and interaction terms were used in generalized linear models to examine the association between elevated cortisol levels and cerebral glucose metabolism as measured by Fluorodeoxyglucose positron emission tomography (FDG-PET) in men and women across Alzheimer's disease spectrum. Sex-stratified analysis was conducted a priori based on established biological differences in HPA axis function between sexes. Elevated cortisol levels were negatively associated with brain glucose consumption in women, but not in men. Women with APOE4 alleles were at greater risk of brain hypometabolism. Diastolic blood pressure in men, but not women, was negatively associated with brain glucose consumption, an indication of higher vascular vulnerability in men. Notably, while sex-stratified analyses revealed these differential patterns, the formal cortisol^*^sex interaction term did not reach statistical significance (*p* = 0.157), potentially due to limited statistical power for detecting interactions. Larger studies are warranted to confirm these sex-specific findings. These findings suggest that cortisol may represent a modifiable risk factor warranting further investigation, particularly in postmenopausal women who experience estrogen decline, a known neuroprotective mediator.

## 1 Introduction

Cerebral glucose metabolism varies significantly between people with normal cognition, mild cognition impairment (MCI), and Alzheimer's disease (AD), which indicates different pathological mechanisms across the AD spectrum (1, 2). Fluorodeoxyglucose positron emission tomography (FDG-PET), specifically radiotracer 18F, had provided crucial insights into the integrity of brain function by measuring cerebral glucose metabolism in the brain (3). A recognizable pattern of low uptake in FDG-PET has been indicative of cognitive decline in AD, this gave FDG-PET a critical role in monitoring AD progression (4–6). Several risk factors such as obesity, diabetes, smoking, and high blood pressure (7) that affect human metabolic function in normally aging individuals are also shared among neurological diseases such as AD (8). Nevertheless, late pathological mechanism of AD involves greater contribution of amyloid-β (Aβ) deposition, neurofibrillary tangles composed of tau protein, and neuronal loss, which showed more pronounced brain hypometabolism and cognitive dysfunction at late stage of AD (5, 9).

Sex differences related to metabolic profiles for men and women, their impact on the aging brain, and the development of neurodegenerative diseases have been investigated previously, but not thoroughly. Compared to men, women exhibited better consumption of glucose in the hippocampus and prefrontal cortex which are associated with memory and decision-making (10). Moreover, Estrogen which is prevalent in women, plays a critical role in regulating glucose consumption through the efficient interaction with GLUT3 and GLUT4, glucose transporters (11). Nevertheless, postmenopausal women showed significant reduction in brain metabolism, which evidently related to the significant reduction of estrogen levels, indicating a steep decline of neuroprotection in women (10). Brain regions affected metabolically with aging in men were medial parietal and frontal cortices areas (12), where in women, posterior cingulate cortex and temporoparietal areas were more affected (10, 12). The pattern of increased risk factors profile such as estrogen reduction at postmenopausal stage, hypometabolism, and greater Aβ deposition might have a compounding effect that had led to greater occurrence of AD in women compared to men (10). Men on the other hand seems to have greater signs of cellular inflammation and hypoperfusion (11). These patterns were mirrored in a recent animal model study, where female mice showed greater brain Aβ deposition, tau protein buildup, and lower brain glucose consumption (13). Where male mice exhibited hypothalamic inflammation that had led to the impairment of brain regular glucose consumption (13). Furthermore, in a recent neuroimaging and genetic study, imaging indicators showed greater metabolic dysfunction in women with progressive AD, but not in men (6). Moreover, four single nucleotide polymorphisms (SNPs) in the fibrinogen gamma chain gene (FGG) were associated with Aβ and tau, which had greater impact on women brain metabolism (6).

Cortisol is a steroid hormone released to combat stress (14). Its role in modulating mitochondrial function, leading to dysfunctional metabolism, is gaining traction in the context of brain health and neurodegeneration (4, 11, 15). One study implied that chronic stress, which would demand constant elevated cortisol, can lead individuals to neurodegenerative diseases like AD (16). This implication was validated in a recent neuroimaging study, where hippocampal hypometabolism was associated with elevated cortisol (15). The same study found that the interaction between elevated cortisol and estrogen affects normal consumption of glucose in women at AD risk (15). Despite these findings, poorer delayed memory predicted by elevated cortisol levels were found in men compared to women in the same study (15). Men with post-traumatic stress disease (PTSD) have shown cellular inflammation presented by oxidative stress and mitochondrial dysfunction which, as a result, impacted their brain metabolism (14). Higher cortisol levels effects were investigated in association with cognition and brain volume in a large dataset with middle aged participants, their findings indicated higher impact of elevated cortisol level on women compared to men (17). Collectively, this evidence suggest that elevated cortisol levels modulate normal brain metabolism, and that women could be at greater risk compared to men, especially at older age.

Importantly, sex differences in hypothalamic-pituitary-adrenal (HPA) axis regulation are well-documented. Women show greater HPA axis reactivity to stress, different cortisol diurnal patterns, and experience more pronounced hormonal changes during aging, particularly following menopause (18, 19). These biological differences provide a strong rationale for examining cortisol effects separately in men and women.

This cross-sectional study aims to investigate the role of cortisol and other cardiovascular risk factors on brain hypometabolism measured by FDG-PET in a subset of data that included participants of Alzheimer's Disease Spectrum. While previous Alzheimer's Disease Neuroimaging Initiative (ADNI) studies have examined cortisol in relation to cerebral glucose metabolism (4) and amyloid deposition (20), neither employed a priori sex-stratified analyses. The present study extends this prior work in three important ways: (1) we conducted sex-stratified analyses planned a priori based on established biological differences in HPA axis function between men and women; (2) we examined a comprehensive set of cardiovascular and metabolic risk factors simultaneously to contextualize cortisol's effects; and (3) we explored whether distinct sex-specific pathways to brain hypometabolism might exist, which could have implications for future sex-tailored risk assessment and intervention strategies pending confirmation in larger studies.

## 2 Methods

The data was derived from the publicly available Alzheimer's Disease Neuroimaging Initiative (ADNI) database at the Laboratory of Neuroimaging (LONI) (https://adni.loni.usc.edu/). ADNI was launched in 2003 to examine the progression of cognition across the AD spectrum using a combination of neuroimaging, psychological assessments, and clinical and biological markers. ADNI participants have provided informed written consent across all recruitment cites as described elsewhere (21), and we have been granted access to the ADNI data archived at LONI. Baseline data of participants demographics such as age, sex, years of education and other factors such as Apolipoprotein E4 epsilon (APOE4) status, Phosphorylated tau (p-tau) levels, intracranial volume, diagnosis of cognition and AD, and FDG-PET measurements were obtained from the “ADNIMERGE” file at LONI. Vital measurements such as systolic and diastolic blood pressure, and a calculated body mass index (BMI) were obtained from the ‘Vital Signs' file at LONI. Diabetes diagnosis was derived from the “ADSP Phenotype Harmonization Consortium (PHC)—Composite Cardiovascular Risk Scores” file, which included a binary variable indicating diagnosis for diabetes. The plasma cortisol serum data was obtained from the “ADMC Duke Biocrates MxP Quant 500 Ultra Performance Liquid Chromatography Longitudinal” file at LONI. The Biocrates MxP Quant 500 Ultra Performance Liquid Chromatography kit was used to extract cortisol serum (μmol/L) from plasma (22). In accordance with the gold standard for steroid hormone analysis, data from this Liquid Chromatography-tandem Mass Spectrometry (LC-MS/MS) platform was selected over data offered by ADNI from available immunoassay-based platforms (e.g., Luminex xMAP) to ensure high molecular specificity and to minimize potential cross-reactivity with other steroid metabolites. Fasting Glucose serum (mmol/l) and serum lipids (mmol/l) (HDL, LDL, and triglycerides) were obtained from “ADMC Nightingale Platform NMR Analysis of Lipoproteins and Metabolites” file at LONI, these serums were extracted using the high-throughput serum nuclear magnetic resonance (NMR) metabolomics platform that measures standard biomarkers concentration (23). Total cholesterol (TC) was then calculated according to the American Heart Association (AHA) guidelines (TC = HDL + LDL + 20% triglycerides (24). Alcohol abuse and smoking status were obtained from the “Medical History” file. Further information regarding the collection and processing procedures of the obtained data can be found elsewhere; ADNI documentations: https://adni.loni.usc.edu/help-faqs/adni-documentation/.

### 2.1 Participants

Eight hundred and twenty-two participants with post-processed FDG-PET output were included in this study. The cognition status varied among participants in the study sample, where 188 participants were cognitively normal (Men = 105, Women = 83), 512 participants were diagnosed with MCI (Men = 286, Women = 226), and 122 participants were diagnosed with AD (Men = 78, Women = 44).

### 2.2 FDG-PET image acquisition

The ADNI FDG-PET protocol was standardized across imaging sites. It ensured reliability and reproducibility of the imaging procedure for cerebral glucose metabolism. Before proceeding with imaging, it was verified that Participants fasted for at least 2 h and blood glucose level was below 180 mg/dL. When 185 MBq (5 mCi) of [18F]-FDG was administered intravenously, participants rested in a room with quiet and dim atmosphere for 20 min to allow tracer uptake in the brain and minimize sensory stimuli. Imaging started 30 min post-injection, dynamic 3D scans or a single 30-min scan for PET/CT systems were acquired, with attenuation correction applied. Scanner-specific parameters were used for image reconstruction, then images were reviewed for quality control before transferring them to the LONI centralized quality control and analysis (25).

The output of post-processed FDG-PET data used in our analyses was extracted from the “ADNIMERGE” file. The primary outcome measure was the Standardized Uptake Value Ratio (SUVR), representing the average FDG-PET uptake of angular, temporal, and posterior cingulate areas (metaROI) as described elsewhere (26). Images were preprocessed using a standardized pipeline including spatial normalization to MNI space and intensity normalization using pons/cerebellar vermis as the reference region, yielding SUVR values that account for individual differences in global tracer uptake. This preprocessing approach is consistent with established ADNI methodology. This composite MetaROI SUVR measure has demonstrated high sensitivity and specificity for detecting AD-related metabolic changes. The raw FDG-PET images can be accessed at LONI in the Image Collections section of ADNI repository.

### 2.3 Statistical analysis

For statistical analyses, Python 3.11 was used with the following packages: statsmodels for GLM analyses, pandas for data manipulation, and scipy for statistical tests. Mean and standard deviation (SD) were used to summarize continuous variables, whereas count and percentage were used to summarize categorical variables. Complete case analysis was employed; participants with missing data on any covariate were excluded from analyses.

Generalized Linear Model (GLM) was conducted to investigate the associations between cardiovascular, hormonal, and genetical risk factors and FDG-PET. The main effect model, our primary model, used a Gaussian family with identity link function: FDG-PET = β_0_ + β_1_ (Age) + β_2_ (Sex) + β_3_ (Education) + β_4_ (ICV) + β_5_ (p-tau) + β_6_ (APOE4) + β_7_ (BMI) + β_8_ (Alcohol) + β_9_ (Smoking) + β_10_ (Cortisol) + β_11_ (SBP) + β_12_ (DBP) + β_13_ (Glucose) + β_14_ (Total Cholesterol) + β_15_ (Diagnosis) + ε. The baseline diagnosis variable was coded as ordinal (CN = 0, MCI = 1, AD = 2). The variance proportion explained by the model in FDG-PET was assessed by the R^2^ value.

To investigate if cortisol effect was moderated by other covariates, we ran separate GLMs between cortisol and other covariates. This was done by adding an interaction term (e.g., cortisol^*^age) to the primary model. The output coefficient and *p*-value from the interaction terms were used to assess the significant of the moderation effect.

Finally, to investigate potential sex-specific effect of cortisol and other covariates on FDG-PET, main effect GLMs were run in men and women stratified subsamples. These sex-stratified analyses were planned a priori based on established biological differences in HPA axis function and cortisol regulation between sexes (18), rather than conducted *post-hoc*. We note that while formal interaction testing (cortisol^*^sex) represents the standard statistical approach to assess effect modification, interaction tests are known to require substantially larger sample sizes than main effect analyses, typically four times the sample size to achieve equivalent power (27, 28). Therefore, we present both formal interaction results and pre-planned stratified analyses, with appropriate interpretation of each.

Multicollinearity was assessed by investigating the Variance Inflation Factor (VIF). VIF was calculated on standardized (z-scored) variables to avoid artificial inflation due to different measurement scales. For each predictor, the effect size and *p*-value (*p*-value < 0.05) outputs were used to assess their significance. Moreover, Sensitivity analyses were conducted to assess robustness of findings.

## 3 Results

The study sample included participants of different cognitive profiles at baseline [CN (cognitively normal), MCI, and AD]. The sample characteristics were summarized in Table 1. A total of 822 participants were included in our analyses, the CN group included 105 men and 83 women (Mean age ± SD); Men = 74.81 ± 6.41, Women = 72.85 ± 5.9, the MCI group included 286 men and 226 women (Mean age ± SD); Men = 72.61 ± 7.003, Women = 71.52 ± 7.53, and the AD group included 78 men and 44 women (Mean age ± SD); Men = 75.72 ± 8.32, Women = 75.58 ± 7.66.

**Table 1:** Characteristics of the study sample (*n* = 822).

**Parameters**	**CN women**	**CN men**	**MCI women**	**MCI men**	**AD women**	**AD men**
N	83	105	226	286	44	78
Age	72.85 ± 5.94	74.82 ± 6.41	71.53 ± 7.53	72.62 ± 7.00	73.72 ± 8.32	75.59 ± 7.66
Education (years)	15.63 ± 2.59	16.99 ± 2.72	15.81 ± 2.64	16.75 ± 2.53	14.66 ± 2.70	16.03 ± 2.73
ICV (mm^3^)	1,431,378.43 ± 132,901.27	1,590,076.57 ± 136,285.01	1,422,458.63 ± 121,440.21	1,608,402.97 ± 144,229.92	1,419,290.00 ± 131,698.56	1,612,656.54 ± 158,241.09
p-tau	22.70 ± 10.29	21.35 ± 8.26	27.84 ± 16.21	25.85 ± 12.16	41.98 ± 18.62	34.70 ± 14.38
BMI	26.72 ± 4.37	27.03 ± 3.41	26.35 ± 4.43	27.09 ± 3.44	24.37 ± 3.86	25.95 ± 3.86
Systolic blood pressure	134.86 ± 16.68	135.50 ± 16.16	136.52 ± 17.26	135.70 ± 15.98	135.05 ± 14.04	137.09 ± 16.90
Diastolic blood pressure	73.86 ± 10.00	76.70 ± 9.51	73.84 ± 9.16	76.38 ± 8.99	75.09 ± 8.76	75.79 ± 8.68
Fasting glucose (mmol/l)	3.92 ± 0.76	4.13 ± 0.73	3.87 ± 0.73	4.29 ± 1.04	3.83 ± 0.56	4.13 ± 0.71
Total cholesterol (mmol/l)	3.16 ± 0.51	2.75 ± 0.48	3.16 ± 0.53	2.83 ± 0.49	3.26 ± 0.55	2.76 ± 0.51
Cortisol (μmol/L)	0.32 ± 0.12	0.28 ± 0.10	0.31 ± 0.12	0.30 ± 0.11	0.36 ± 0.12	0.34 ± 0.12
Diabetes (No/Yes)	78/5	90/15	207/19	247/39	41/3	70/8
Smoking (No/Yes)	54/29	59/46	144/82	176/110	33/11	41/37
Alcohol abuse (No/Yes)	83/0	98/7	220/6	271/15	44/0	73/5
APOE4 (0/1/2 alleles)	61/21/1	77/24/4	126/80/20	143/109/34	11/24/9	26/30/22

The continuous variables are presented as mean±standard deviation, whereas the categorical variables are presented as count. CN, Cognitively Normal; MCI, Mild Cognitive Impairment; AD, Alzheimer's disease; ICV, intracranial volume; p-tau, phosphorylated tau protein; APOE, apolipoprotein E; BMI, body mass index; FDG-PET, fluorodeoxyglucose (FDG)-positron emission tomography (PET).

### 3.1 The effects of vascular and hormonal risk factors on cerebral glucose metabolism

**Figure 1:**
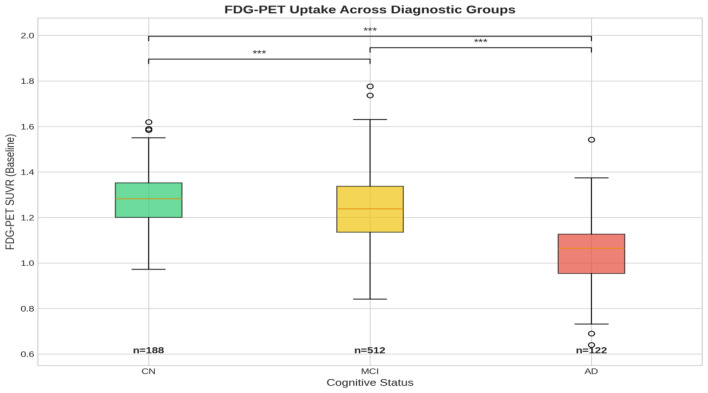
A boxplot of the three diagnostic groups at baseline and their baseline FDG-PET uptake. CN, Cognitively Normal; MCI, Mild Cognitive Impairment; AD, Alzheimer's disease; FDG-PET, fluorodeoxyglucose (FDG)-positron emission tomography (PET).

The association between vascular/hormonal factors and FDG-PET outcomes are presented in Table 2. Age (β = −0.0032, 95% CI: −0.0046 to −0.0018, *p* < 0.001), cortisol (β = −0.1147, 95% CI: −0.201 to −0.028, *p* = 0.009), p-tau (β = −0.0017, *p* < 0.001), APOE4 (β = −0.0179, *p* = 0.021), alcohol abuse (β = −0.0536, *p* = 0.030), diastolic blood pressure (β = −0.0013, *p* = 0.025), and fasting glucose (β = −0.0207, *p* < 0.001) were negatively associated with cerebral glucose metabolism measured by FDG-PET. The cognition diagnostic status at baseline (CN to MCI to AD) showed negative association with FDG-PET measurements (β = −0.0859, *p* < 0.001), reflecting a sharp decline in cerebral glucose consumption across these groups (Figure 1). The model-fit analysis showed that the predictors in the main effect GLM explained 28.1% of FDG-PET uptake variability (R^2^ = 0.281). All standardized VIF values ranged from 1.05 to 1.69, indicating acceptable levels of multicollinearity [VIF < 5 is generally considered acceptable; (29)], see Supplementary Table S1-A.

**Table 2:** The associations between all covariates and FDG-PET in the generalized linear regression model for the whole sample.

**Risk Factors**	**β coefficient**	**95% CI**	**SE**	***p*-value**
**Age**	**−0.0032**	[−0.0046, −0.0018]	**0.0007**	**< 0.001** ^*^
Sex	−0.0184	[−0.0427, 0.0059]	0.0124	0.138
Education	0.0013	[−0.0023, 0.0049]	0.0019	0.484
ICV	−0.0000	[−0.0000, 0.0000]	0.0000	0.773
**p-tau**	**−0.0017**	[−0.0025, −0.0010]	**0.0004**	**< 0.001** ^*^
**APOE4**	**−0.0179**	[−0.0332, −0.0027]	**0.0078**	**0.021** ^*^
BMI	0.0009	[−0.0017, 0.0035]	0.0013	0.479
**Alcohol Abuse**	**−0.0536**	[−0.1019, −0.0053]	**0.0247**	**0.030** ^*^
Smoking	0.0044	[−0.0153, 0.0240]	0.0100	0.665
**Cortisol**	**−0.1147**	[−0.2011, −0.0283]	**0.0441**	**0.009** ^*^
Systolic blood pressure	−0.0001	[−0.0008, 0.0005]	0.0003	0.669
**Diastolic blood pressure**	**−0.0013**	[−0.0025, −0.0002]	**0.0006**	**0.025** ^*^
**Fasting glucose**	**−0.0207**	[−0.0320, −0.0093]	**0.0058**	**< 0.001** ^*^
Total cholesterol	0.0022	[−0.0170, 0.0213]	0.0098	0.826
**Baseline diagnosis**	**−0.0859**	[−0.1024, −0.0695]	**0.0084**	**< 0.001** ^*^

^*^Significant findings (*p*-value < 0.05) are marked with asterisk and bold. 95% CI, Confidence Interval; ICV, intracranial volume; p-tau, phosphorylated tau protein; APOE, apolipoprotein E; BMI, body mass index; FDG-PET, fluorodeoxyglucose (FDG)-positron emission tomography (PET).

### 3.2 The interaction between cortisol and vascular/hormonal risk factors on cerebral glucose metabolism

This analysis was conducted to investigate whether the effect of cortisol shown in the main effect model was moderated by other covariates. We added an interaction term between cortisol and each covariate in the main effect GLM, running these models separately while controlling for other covariates. None of the interaction terms reached statistical significance (Table 3), indicating a strong and independent effect of cortisol on cerebral glucose metabolism. Importantly, the cortisol^*^sex interaction term did not reach statistical significance (β = 0.1201, *p* = 0.157), indicating that the data do not provide formal statistical evidence that the cortisol-FDG relationship differs between men and women. However, given the known power limitations of interaction tests and the strong a priori biological rationale for sex differences in HPA axis function, we proceeded with the pre-planned stratified analyses as exploratory investigations.

**Table 3:** The interaction between plasma cortisol levels and the covariates in separate generalized linear regression models for the whole sample.

**Risk factors**	**β coefficient**	***p*-value**
Cortisol^*^Age	−0.0014	0.809
Cortisol^*^Sex	0.1201	0.157
Cortisol^*^p-tau	0.0040	0.174
Cortisol^*^APOE4	0.0357	0.532
Cortisol^*^BMI	−0.0076	0.484
Cortisol^*^Alcohol Abuse	0.0019	0.994
Cortisol^*^Smoking	0.1210	0.170
Cortisol^*^Systolic blood pressure	0.0022	0.443
Cortisol^*^Diastolic blood pressure	0.0045	0.326
Cortisol^*^Fasting glucose	−0.0028	0.961
Cortisol^*^Total cholesterol	−0.0313	0.716
Cortisol^*^Baseline Diagnosis	−0.0642	0.350

*p*-value < 0.05 indicates statistically significant findings from the generalized linear regression model. ICV, intracranial volume; p-tau = phosphorylated tau protein; APOE = apolipoprotein E; BMI = body mass index; FDG-PET = fluorodeoxyglucose (FDG)-positron emission tomography (PET).

### 3.3 Stratified analysis by sex

Despite the non-significant cortisol^*^sex interaction, we proceeded with our pre-planned sex-stratified analyses based on the strong a priori biological rationale for examining cortisol effects separately in men and women, given well-documented sex differences in HPA axis function (18). These stratified analyses should be interpreted as exploratory. The stratified analysis by sex showed clearer view on the mechanism of the association between vascular/hormonal risk factors and cerebral glucose metabolism (Table 4). In women, age (β = −0.0039, *p* = 0.001), cortisol (β = −0.1701, 95% CI: −0.301 to −0.039, *p* = 0.011), p-tau (β = −0.0016, *p* = 0.004), APOE4 (β = −0.0329, *p* = 0.020), fasting glucose (β = −0.0292, *p* = 0.008), and the diagnosis of cognition status (β = −0.0777, p < 0.001) remained significant and negatively associated FDG-PET, but not diastolic blood pressure (β = −0.0001, *p* = 0.874). In men, age (β = −0.0029, *p* = 0.002), p-tau (β = −0.0017, *p* = 0.002), diastolic blood pressure (β = −0.0024, 95% CI: −0.0039 to −0.0008, *p* = 0.003), fasting glucose (β = −0.0146, *p* = 0.033), and the diagnosis of cognition status (β = −0.0927, *p* < 0.001) remained significant and negatively associated with FDG-PET, but not, cortisol (β = −0.0589, *p* = 0.324) and APOE4 (β = −0.0118, *p* = 0.209). The variance explained by the GLM in women for FDG-PET uptake was 33.25% (R^2^ = 0.3325) and 30.99% (R^2^ = 0.3099) in the GLM for men.

**Table 4:** The association between all covariates and FDG-PET in a stratified sample by sex.

**Risk factors**	**Women β coef**	**Women 95% CI**	**Women *p*-value**	**Men β coef**	**Men 95% CI**	**Men *p*-value**
**Age**	**−0.0039**	[−0.006, −0.002]	**< 0.001** ^*^	**−0.0029**	[−0.005, −0.001]	**0.002** ^*^
Education	0.0038	[−0.002, 0.010]	0.202	−0.0010	[−0.006, 0.004]	0.672
ICV	0.0000	[−0.000, 0.000]	0.563	−0.0000	[−0.000, 0.000]	0.410
**p-tau**	**−0.0016**	[−0.003, −0.000]	**0.004** ^*^	**−0.0017**	[−0.003, −0.001]	**0.002** ^*^
**APOE4**	**−0.0329**	[−0.061, −0.005]	**0.020** ^*^	−0.0118	[−0.030, 0.007]	0.209
BMI	0.0008	[−0.003, 0.005]	0.692	0.0016	[−0.002, 0.005]	0.401
Alcohol Abuse	−0.0856	[−0.202, 0.031]	0.149	−0.0473	[−0.100, 0.006]	0.079
Smoking	0.0195	[−0.012, 0.051]	0.231	−0.0118	[−0.037, 0.014]	0.362
**Cortisol**	**−0.1701**	[−0.301, −0.039]	**0.011** ^*^	−0.0589	[−0.176, 0.058]	0.324
Systolic blood pressure	−0.0006	[−0.002, 0.000]	0.259	0.0004	[−0.000, 0.001]	0.391
Diastolic blood pressure	−0.0001	[−0.002, 0.002]	0.874	**−0.0024**	[−0.004, −0.001]	**0.003** ^*^
**Fasting glucose**	**−0.0292**	[−0.051, −0.008]	**0.008** ^*^	**−0.0146**	[−0.028, −0.001]	**0.033** ^*^
Total cholesterol	−0.0038	[−0.033, 0.025]	0.796	0.0101	[−0.016, 0.036]	0.443
**Baseline diagnosis**	**−0.0777**	[−0.104, −0.051]	**< 0.001** ^*^	**−0.0927**	[−0.114, −0.072]	**< 0.001** ^*^

^*^Significant findings (*p-*value < 0.05) are marked with asterisk. 95% CI, Confidence Interval; ICV, intracranial volume; p-tau, phosphorylated tau protein; APOE, apolipoprotein E; BMI, body mass index; FDG-PET, fluorodeoxyglucose (FDG)-positron emission tomography (PET).

The effect size of cortisol in women (β = −0.1701) remained the highest in the GLM for women, which indicates strong hormonal influence on the cerebral glucose metabolism in women, where in men the highest effect size among the risk factors was fasting glucose (β = −0.0146). The significant negative associations show that when predictors levels increase, cerebral glucose metabolism decreases.

### 3.4 Sensitivity analyses

To examine potential dose-response relationships, cortisol was categorized into tertiles. Compared to the lowest tertile, participants in the highest cortisol tertile showed significantly lower FDG-PET SUVR (β = −0.041, *p* = 0.030), with an intermediate effect in the middle tertile (β = −0.023, *p* = 0.189), supporting a dose-response pattern, results are presented in Supplementary Table S1B. Among women, subgroup analysis by APOE4 status revealed that APOE4 carriers showed a numerically stronger cortisol-FDG association (β = −0.227) compared to non-carriers (β = −0.142), though this difference did not reach statistical significance (interaction *p* = 0.31), likely due to reduced sample size in subgroups. Model robustness was confirmed by comparing full vs. reduced models. Key findings remained stable across model specifications: cortisol effect in women [reduced model (β = −0.168); full model (β = −0.170)], APOE4 effect, and the sex-specific pattern of associations.

## 4 Discussion

The associations between vascular/hormonal associations with cerebral glucose metabolism measured by FDG-PET was investigated in an AD spectrum sample from ADNI. At baseline, age, sex, cortisol, education, ICV, p-tau, APOE4, BMI, alcohol abuse, smoking, systolic blood pressure, diastolic blood pressure, fasting glucose, total cholesterol, and baseline diagnosis were the main predictors of cerebral hypometabolism. Despite the complexity of the mechanisms of the cerebral glucose metabolism, the model-fit results (28.1%) explained substantial portion of the variance in FDG-PET. While this explained variance may appear modest, it is consistent with the complex, multifactorial nature of brain metabolism and comparable to prior neuroimaging studies examining similar relationships (4). However, when the sample was stratified by sex to examine these associations from biological and hormonal differences perspective, cortisol and APOE4 where specifically associated with women, but not in men. While diastolic blood pressure was specifically associated with men, but not in women. Age, p-tau, fasting glucose, and diagnosis remained significant for both men and women. Alcohol abuse lost its significant association with FDG-PET in the stratified analysis, but the negative pattern was stronger in men, but not in women.

Our findings extend prior ADNI cortisol studies by employing sex-stratified analyses based on established biological differences in HPA axis function. In these exploratory stratified models, cortisol was significantly associated with brain hypometabolism only in women, while diastolic blood pressure showed significant associations only in men. However, we emphasize that the cortisol^*^sex interaction did not reach statistical significance (*p* = 0.157), these observed differences in stratified analyses do not constitute formal statistical evidence that sex modifies the cortisol-FDG relationship. The patterns observed, hormonal factors appearing stronger in women and vascular factors in men, align with known biological differences and generate hypotheses for future sex-specific investigations of AD risk assessment.

Elevated cortisol levels prior to symptomatic AD have been linked to worse outcomes and accelerated cognitive decline in the literature (30–32). To our knowledge, most imaging studies to date have focused on extracted volumes of brain MRI images, primarily targeting the hippocampus and limbic regions (33). These regions are critical for hormonal regulation in the human body and are highly susceptible to neuronal damage in AD. However, glucocorticoid receptors (GRs) are present in multiple cortical brain regions, which were found to be susceptible as well in the pathological mechanism of AD (15, 34, 35). The association between elevated cortisol levels and elevated Aβ deposition was found in functional studies using PET imaging, their findings where present across the AD spectrum (20, 36). Cognitive decline was linked to elevated cortisol levels that were mediated by brain volume. The mediation effect on cognition was also suggested between cortisol and Aβ in the elderly (37, 38).

Cortisol sex-specific effect was discussed in an AD risk middle aged smaller sample (15). Their study reported significant effect of elevated cortisol levels on the cerebral metabolic rates of glucose (CMRglc) in middle frontal brain regions in postmenopausal women (15). Our results support their findings, where only women showed significant and independent association with brain hypometabolism measured by FDG-PET. The mean age for women in our sample was over 70 years old across the diagnostic groups and they were assumed postmenopausal. When compared to the age of their women sample, it was significantly higher than their postmenopausal women (*n* = 88, age mean ± SD = 56 ± 5). Taken together, these findings emphasize the importance of clinical management of elevated cortisol levels due to the sharp neuroprotection decline associated with sharply declined estrogen levels in postmenopausal women (10, 15). Importantly, estrogen levels were not directly measured in this study, and menopausal status was inferred based on participant age (mean age ~73 years). Therefore, the proposed role of estrogen decline in mediating cortisol's effects on brain metabolism remains a hypothesis requiring validation in studies with direct hormonal measures. In men, no associations were found between elevated cortisol levels and cerebral glucose consumption in our investigation. However, other reports showed evidence regarding its impact on Adenosine triphosphate (ATP) production, cortical volume and increased mitochondrial oxidative stress in men (14, 15). Moreover, several other potential biological explanations for the sex-specific cortisol effect observed in our study. Women demonstrate greater HPA axis reactivity and prolonged cortisol responses to psychosocial stress compared to men (18). This heightened responsivity may result in greater cumulative glucocorticoid exposure and subsequent metabolic effects. Also, the decline in estrogen following menopause removes a protective factor against cortisol's neurotoxic effects, as estrogen modulates glucocorticoid receptor expression and buffers against stress-induced hippocampal damage (39). The postmenopausal status of most women in our sample (mean age ~73 years) may render them particularly vulnerable. Another potential biological explanation is the sex differences in brain glucose transporter expression (GLUT1, GLUT3) which may influence susceptibility to cortisol-induced metabolic disruption (15).

Other independent sex-specific effects beyond the scope of this study were found in our analysis. Table 1 showed the number of participants in each diagnostic group with APOE4 status (alleles carriers), women carrying APOE4 isoforms were negatively associated with brain hypometabolism but not in men carrying APOE4. This sex-specific APOE effect is consistent with epidemiological evidence that APOE4 confers greater AD risk in women than men. The trend toward stronger cortisol effects in female APOE4 carriers suggests a potential gene-hormone interaction warranting further investigation, especially when it was reported that women carrying APOE4 were more susceptible to accelerated cognitive decline and decreased hippocampal volume compared to men (40, 41). In men, we found an independent association between diastolic blood pressure and brain hypometabolism. This finding suggests sex-specific vascular contributions to brain metabolism, with men showing greater susceptibility to blood pressure related cerebrovascular effects. The pattern of association between blood pressure and AD was strictly identified in systolic blood pressure in the literature (42). Nevertheless, altered pulse pressure in normal aging is caused by arterial stiffness, a mechanism which the blood vessel walls thickens (43). This mechanism increases systolic blood pressure and reduces diastolic blood pressure, which widens the pulse pressure, resulting in excessive damage to microvasculature organs like the brain (44, 45). Our results suggest that men have higher vascular vulnerability, as suggested as well elsewhere (11), compared to women. These findings encourage future sex-specific investigations.

There are several limitations that should be considered when interpreting our findings. Most importantly, while our sex-stratified analyses revealed different patterns of association to brain hypometabolism between men and women (cortisol significant only in women; diastolic blood pressure significant only in men), the cortisol^*^sex interaction term did not reach statistical significance (*p* = 0.157). Therefore, these stratified findings should be interpreted as exploratory rather than confirmatory of sex moderation effect to the association between cortisol and brain metabolism. Interaction tests typically require substantially larger sample sizes than main effect analyses to achieve adequate power (27), and our study may have been underpowered to detect such effects. Future studies with larger sample size specifically powered for interaction testing are needed to formally determine whether sex moderates the association between cortisol and brain metabolism. The cross-sectional nature of our analysis prevents causality interpretations but emphasizes the need for clinical consideration and monitoring of the significant findings. Moreover, we acknowledge that the MetaROI primarily captures temporoparietal and posterior cingulate regions, which may be more sensitive to changes typically observed in women, while potentially underrepresenting frontal and medial parietal changes more characteristic of male metabolic decline patterns (12). The use of single time-point plasma cortisol rather than integrated measures such as hair cortisol, multiple daily samples and 24-h urinary cortisol may not fully capture chronic cortisol exposure. However, fasting morning cortisol has been validated as a meaningful biomarker in AD research and shows reasonable test-retest reliability (46). Furthermore, we did not have direct measures of estrogen or menopausal status. While the mean age of women in our sample (~73 years) strongly suggests postmenopausal status, the mechanistic role of estrogen decline in the observed cortisol-metabolism relationship remains inferential and requires confirmation in studies with comprehensive hormonal profiling. In our analysis, we adjusted for several confounders, but our models only explained about 28%−33% of variance in cerebral glucose metabolism measured by FDG-PET. This should encourage scientists to validate our findings in longitudinal studies to track dynamic changes of cortisol associations with brain metabolism and expand the scope to exam other risk factors to provide further insights into the mechanism of brain metabolism in the AD spectrum population. Despite the well standardized ADNI cohort, our subsample included older participants with mostly cognitive decline diagnosis, therefore, our findings should not be generalized across “healthier” age categories.

## 5 Conclusion

This study highlights the impact of elevated cortisol levels on cerebral glucose metabolism measured by FDG-PET. Women were at higher risk of hypometabolism which should warrant further investigation into this association in future studies to validate the findings of the sex-stratified analyses based on established biological differences in HPA axis function. The sex-specific nature of these findings underscores the importance of considering biological sex in AD biomarker research and suggests potential for sex-tailored intervention approaches. Future longitudinal studies with direct hormonal measures are needed to determine whether cortisol management strategies could benefit postmenopausal women at risk for cognitive decline.

## Data Availability

The original contributions presented in the study are included in the article/Supplementary material, further inquiries can be directed to the corresponding author.

## Supplementary material

Supplementary Figure S1 Scatterplots showing the relationship between plasma cortisol levels and FDG-PET SUVR, stratified by sex. **Left panel**: Women (*n* = 353); **Right panel**: Men (n = 469). Regression lines with 95% confidence bands are shown.

Supplementary Figure S2 Forest plot showing standardized regression coefficients with 95% confidence intervals for all predictors in the main model.

Supplementary Figure S3 Distribution of plasma cortisol levels by sex. Histograms show the distribution of cortisol values for women and men. Vertical dashed lines indicate tertile boundaries.

Supplementary Figure S4 Scatterplot showing the relationship between diastolic blood pressure (DBP) and FDG-PET SUVR in men, demonstrating the significant sex-specific vascular association (β = −0.0024, *p* = 0.003).
