# Supplementary material for: Sex-specific effect of cortisol on cerebral glucose metabolism across Alzheimer's disease spectrum: a neuroimaging study
Source: Front Neurol. 2026 Mar 12;17:1680116. doi: 10.3389/fneur.2026.1680116 (PMC13017249; doi:10.3389/fneur.2026.1680116)
Supplement: Supplementary Figure S1 — Scatterplots showing the relationship between plasma cortisol levels and FDG-PET SUVR, stratified by sex. Left panel: Women (n = 353); Right panel: Men (n = 469). Regression lines with 95% confidence bands are shown. [file Supplementary_file_1.docx]

# Supplementary Materials

## Supplementary Table S1. Sensitivity Analyses Results

**A. Variance Inflation Factors (Standardized Variables)**

| **Variable** | **VIF (Standardized)** | **Assessment** |
| --- | --- | --- |
| Age | 1.40 | Acceptable |
| Sex | 1.14 | Acceptable |
| Education | 1.15 | Acceptable |
| ICV | 1.12 | Acceptable |
| p-tau | 1.16 | Acceptable |
| APOE4 | 1.05 | Acceptable |
| BMI | 1.17 | Acceptable |
| Alcohol | 1.07 | Acceptable |
| Smoking | 1.06 | Acceptable |
| Cortisol | 1.12 | Acceptable |
| SBP | 1.69 | Acceptable |
| DBP | 1.60 | Acceptable |
| Glucose | 1.15 | Acceptable |
| Total Cholesterol | 1.13 | Acceptable |
| Diagnosis | 1.27 | Acceptable |

*Note: VIF calculated on z-score standardized variables. All VIF values < 2.0 indicate acceptable levels of multicollinearity. VIF < 5 is generally considered acceptable (Hair et al., 2019).*

**B. Cortisol Tertile Analysis**

| **Cortisol Tertile** | **β Coefficient** | **SE** | **p-value** |
| --- | --- | --- | --- |
| Low (Reference) | — | — | — |
| Middle vs. Low | -0.023 | 0.017 | 0.189 |
| High vs. Low | -0.041 | 0.019 | **0.030*** |

Low tertile: n=120, mean cortisol=0.200 µmol/L, mean FDG-PET=1.278; Medium tertile: n=115, mean cortisol=0.303 µmol/L, mean FDG-PET=1.245; High tertile: n=118, mean cortisol=0.452 µmol/L, mean FDG-PET=1.196

# Supplementary Figure Legends:


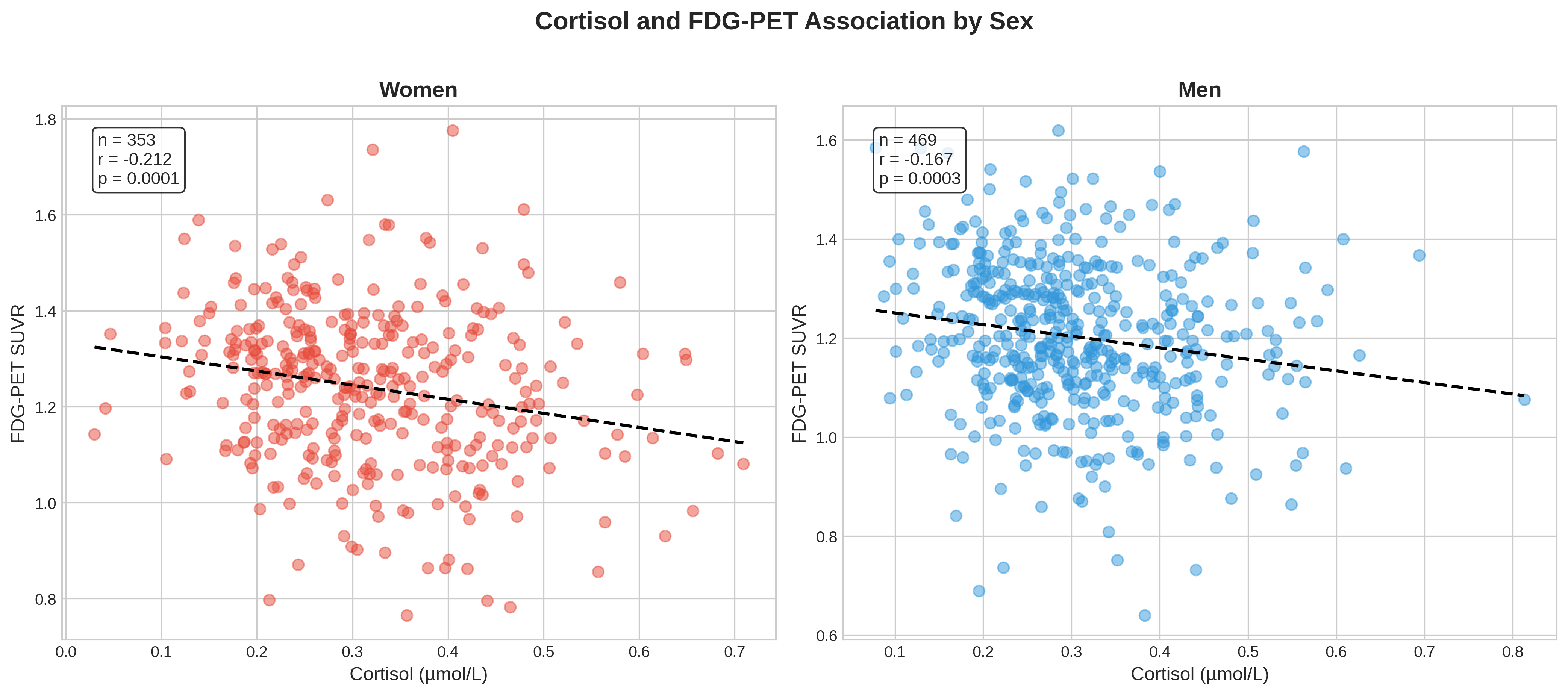
**Supplementary Figure S1.** Scatterplots showing the relationship between plasma cortisol levels and FDG-PET SUVR, stratified by sex. Left panel: Women (n = 353); Right panel: Men (n = 469). Regression lines with 95% confidence bands are shown.


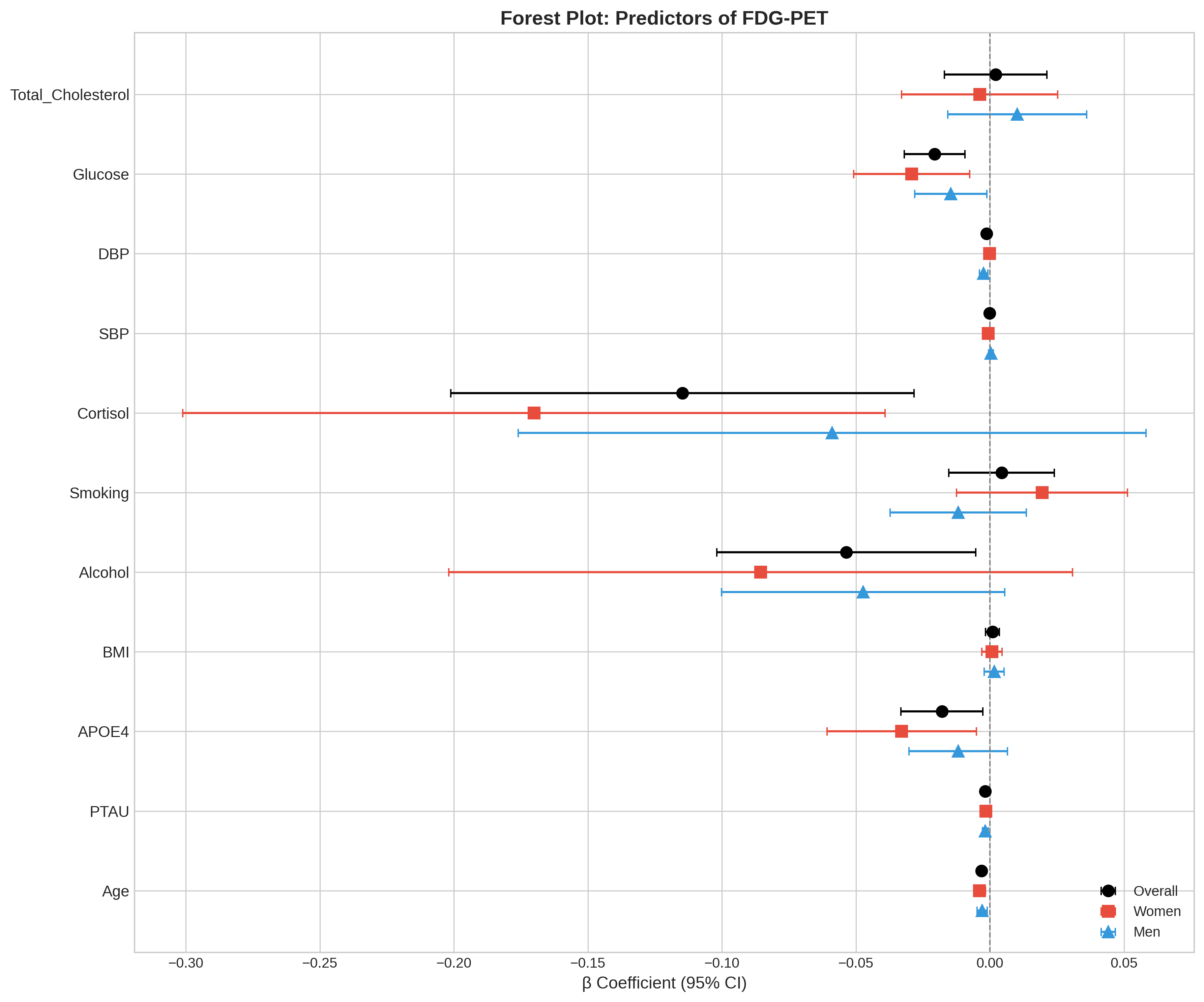
**Supplementary Figure S2.** Forest plot showing standardized regression coefficients with 95% confidence intervals for all predictors in the main model.


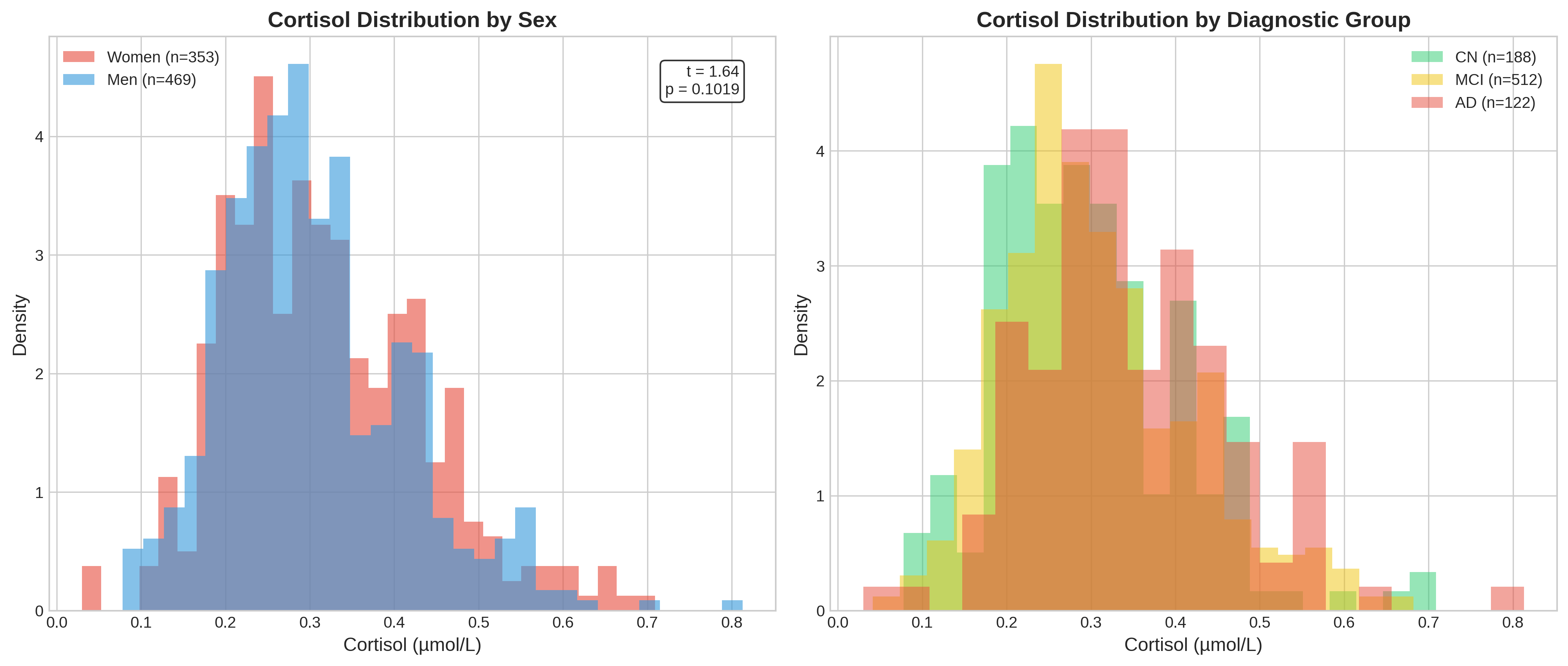
**Supplementary Figure S3.** Distribution of plasma cortisol levels by sex. Histograms show the distribution of cortisol values for women and men. Vertical dashed lines indicate tertile boundaries.


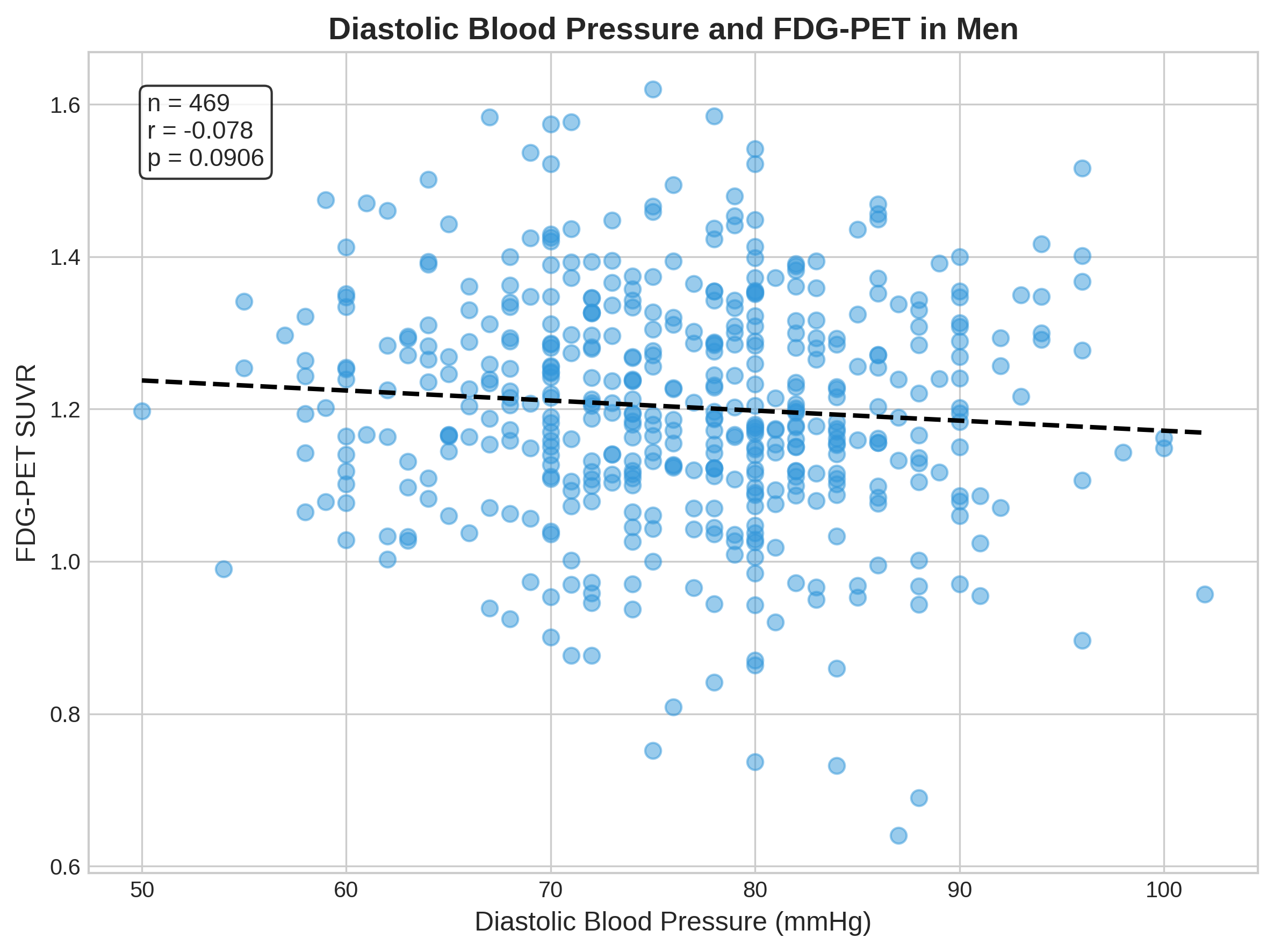


**Supplementary Figure S4.** Scatterplot showing the relationship between diastolic blood pressure (DBP) and FDG-PET SUVR in men, demonstrating the significant sex-specific vascular association (β = -0.0024, p = 0.003).
